# A Low Cost, Novel Treatment of Severe Diabetic Gastroparesis Based on Burkitt’s Dietary Fiber Hypothesis

**DOI:** 10.7759/cureus.18062

**Published:** 2021-09-17

**Authors:** J Wesley Jones, Katrina L Lamont, Jennifer N Stoltenberg, Grace D Brannan

**Affiliations:** 1 Department of Internal Medicine, Jerry M. Wallace School of Osteopathic Medicine, Campbell University, Buies Creek, USA; 2 Department of Internal Medicine, McLaren Macomb Hospital, Mt. Clemens, USA

**Keywords:** abdominal distension, treatment, abdominal bloating, constipation, dietary fiber, gastroparesis, intestinal dysmotility, gastric emptying, intestinal transit

## Abstract

We report a seven-year follow-up of a 43-year-old Hispanic female with severe diabetic gastroparesis (GP) and a 42.5 kg weight loss (45% of body mass), who required feeding jejunostomy tube placement. The patient had an excellent response to a treatment regime directed at increasing stool bulk, enhancing gut transit, and mobilizing intestinal gas by using dietary fiber supplements and osmotic laxatives with as needed tap water enemas. Hospital cost savings for this patient exceeded $125,000 annually. This case study suggests that constipation may substantially contribute to GP.

## Introduction

Gastroparesis (GP) is considered a primary gastric motor disorder without mechanical outlet obstruction. Treatment guidelines include dietary interventions (such as low fat and low roughage meals), nutritional support and correction of electrolytes if indicated, and symptom relief with promotility and anti-emetic medications [1-2]. More severe GP may require a feeding jejunostomy, gastric electrical stimulation, or gastric peroral endoscopic myotomy [3-6]. However, these procedures are inconsistently effective. Furthermore, a recent report found that gastric peroral endoscopic myotomy for GP had a 12.9% annual decline in efficacy among those patients with an initial response [6].

Importantly, three studies demonstrate intestinal dysmotility occurs in most GP patients, which reflects that GP may be part of a global intestinal motor disorder [7-9]. Also, abdominal bloating, distention, and constipation are prevalent in these patients and correlate with GP symptom severity [5, 10-11]. Further, suppressing defecation voluntarily can impair gastric emptying (GE) in healthy subjects [12]. Of note, Denis Burkitt and others linked dietary fiber and bulky stools with rapid intestinal transit [13-15]. Speedy transfer of food waste through the gut should improve gut motility. These observations suggest that constipation may significantly contribute to GP illness by its effects on gut motility.

This case was presented as an e-poster titled “A low cost, novel treatment approach for severe diabetic gastroparesis based on Burkitt’s dietary fiber hypothesis: a case report” at the American College of Gastroenterology 2020 Virtual Annual Scientific Meeting, October 23-28, 2020. The abstract of our e-poster was published in the American Journal of Gastroenterology, October 2020, volume 115, http://journals.lww.com/ajg/Fulltext/2020/10001/S2054_A_Low_Cost,_Novel_Treatment_Approach_for.2054.aspx; doi 10.14309/01.ajg.0000710264.98795.0d. 

## Case presentation

We report the case of a 43-year-old Hispanic female with type 2 diabetes who was referred for intractable nausea, vomiting, and weight loss. 

In 2011, the patient had an esophagogastroduodenoscopy (EGD) to evaluate epigastric pain and reflux. Hiatal hernia, reflux, and a small amount of gastric food residue were noted. GE result was calculated by using extrapolated data and showed gastric half emptying time (T- ½) was 70 min (abnormal: > 90 min). 

In the fall of 2012 (six months prior to the current admission), the patient was admitted for hematemesis, nausea, and vomiting. EGD showed a Los Angeles (LA) Grade D reflux esophagitis, mild gastroduodenitis, and gastric food residue [16].

One month later at the gastroenterology clinic in follow-up for persistent nausea and vomiting, a 9 kg weight loss was recorded. Her weight was 85 kg, and the physical examination was unremarkable. A repeat GE study showed the T-½ was 101 min. However, the patient only ingested one-fourth of the radioactive egg sandwich. GP was diagnosed.

Subsequently, in 2013, the patient had four consecutive hospital admissions within a three-month timeframe, including the current admission for nausea, vomiting, and weight loss. The first admission was complicated by worsening renal failure and *Clostridium difficile* colitis. Laboratory results are shown below (Table 1 ). Metronidazole was given, and hemodialysis was initiated. 

**Table 1:** Laboratory Results Obtained on Admissions During 2013 and the Most Recent Admission in Late 2019 A1C: glycated hemoglobin; BUN: blood urea nitrogen; CO_2_: carbon dioxide; CRP: C-reactive protein; SGOT: serum glutamic oxaloacetic transaminase; SGPT: serum glutamic pyruvic transaminase; WBC: white blood cells

Laboratory	Admission Time Frame						Normal Values
	1st Admission 2013	2nd Admission 2013	3rd Admission 2013	4th Admission 2013	5th Admission 2013	2019 Admission	
Sodium	146	146	148	145	144	135	136 - 145 mmol L
Potassium	4.3	2.9	2.6	4.1	3.2	5.2	3.5 - 5.1 mmol L
Chloride	111	103	106	101	107	99	98 - 107 mmol L
CO_2_	25	31	28	31	22	27	21 - 32 mmol L
BUN	37	16	21	31	20	36	7 - 18 mg dL
Creatinine	5.2	3.4	4.5	9.2	3.9	9.8	0.55 - 1.30 mg dL
SGOT	25	14	10	20	17	57	15 - 37 U L
SGPT	25	12	15	52	18	25	12 - 78 U L
Alkaline phosphatase	54	70	56	75	89	91	45 - 117 U L
Total protein	5.2	5.8	4.7	5.7	5.5	8	6.4 - 8.2 g dL
Albumin	1.2	1.6	1.5	1.6	2.4	3.2	3.4 - 5.0 g dL
Bilirubin, total	0.3	0.4	0.3	0.4	0.2	0.6	0.2 - 1.0 mg dL
Calcium	7.4	8	7.4	7.8	8.4	8.2	8.5 - 10.1 mg dL
Magnesium	1.6	1.5	1.8	2	1.8		1.8 - 2.4 mg dL
Phosphorous	3.1		2.8	4.7	3.3		2.5 - 4.9 mg dL
Glucose	95	117	59	164	94	245	74 - 106 mg dL
WBC	7.4	12.8	7.3	14.4	5.6	6.5	4.5 - 12.5 x10*3 uL
Hemoglobin	8.1	10.8	9.9	15.1	11.2	14.7	12.0 - 16.0 g dL
Platelets	168	287	293	272	232	236	150 - 450 x10*3 uL
Hemoglobin A1C	4.5	4.9					4.2 - 5.8%
Ferritin	595 ng/mL						11 - 307 mcg L
CRP		3.6					0.0 - 2.9 mg L
Amylase		29		25			25 - 115 U L
Lipase		93		121			73 - 393 U L
Lactic acid		1.8			1		0.4 - 2.0 mmol L

During the third admission, her gastroenterologist was consulted. No additional imaging studies were advised. Erythromycin suspension, 400 mg four times daily, was given. The hemodialysis was changed to peritoneal dialysis, and the patient was discharged. The patient was then readmitted within three weeks for persistent nausea and vomiting. Our gastroenterology group was consulted for a second opinion.

The past medical history included hypertension, coronary artery stent placement (2009), a remote history of diabetic ketoacidosis, and cholecystectomy (2009). There was no history of alcohol, tobacco, or illicit drug use. Family history was noncontributory. 

Medications included esomeprazole, 40 mg twice daily; metoclopramide, 5 mg two times daily; promethazine, 25 mg every four hours as needed; ondansetron, 8 mg twice daily; carvedilol, 3.125 mg twice daily; diltiazem 180 mg twice daily; zolpidem, 10 mg at night; sodium bicarbonate, 650 mg daily; Renal Cap, one daily; atorvastatin, 10 mg twice daily; calcium carbonate, 1,000 mg at bedtime; chlorpromazine, 10 mg every four hours as needed; and midodrine, 5 mg twice daily.

The vital signs were stable. The average predialysis weight was 51.5 kg. A flat affect was noted; otherwise, the physical examination was unremarkable. EGD showed an LA grade D reflux esophagitis with a 10 cm length confluent esophageal ulceration extending cephalad from the gastroesophageal junction. Biopsies confirmed esophageal ulcer but were negative for viral pathogens, *Candida*, *H. pylori*, and celiac disease. Abdominal and pelvic computed tomography scans, as well as a small bowel imaging series, were negative. The calorie count was less than 100 calories per day.

Hemodialysis was reinstituted, and a feeding jejunostomy tube was placed. The patient had received peritoneal dialysis for 34 days. Also, 1,000 mL warm water enemas were given, and 17 g polyethylene glycol (PEG) 3350 mixed with 3 g of partially hydrolyzed guar gum (Nutrisource®) were given daily through a jejunostomy tube. Residuals were carefully monitored to avoid possible aspiration. Discharge medications included erythromycin, 250 mg every eight hours, and pantoprazole, 40 mg daily. The esomeprazole was discontinued and discharge medications were otherwise unchanged. The patient also continued fiber supplements and PEG 3350 at discharge and enemas as often as twice weekly for GP symptom control.

Two months after discharge, the patient was admitted for a change in mental status, nausea, and vomiting. An adverse reaction to narcotic medication was suspected.

There were no further admissions in 2013. The jejunostomy tube was removed within four months of insertion. A fasting serum gastrin level in October 2014 to rule out possible gastrinoma was 376. 

Below is a bar graph summarizing the patient’s 23 GP-related hospital admissions from 2013 through 2019.

**Figure 1:**
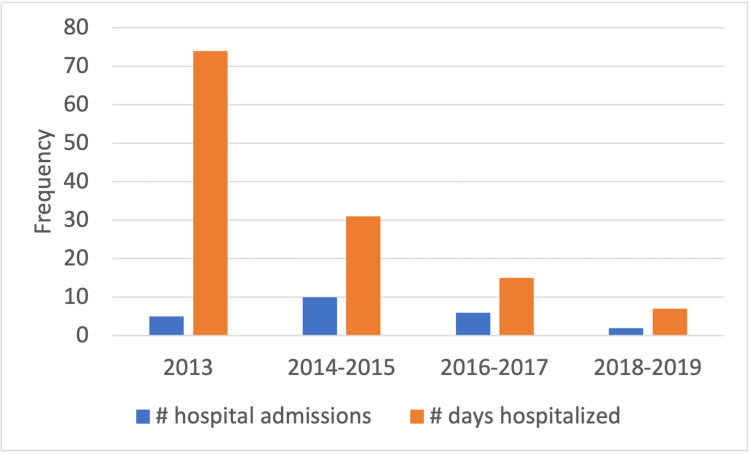
Summary of the patient’s 23 gastroparesis-related hospital admissions from 2013 through 2019

In 2019, the patient was admitted for nausea, vomiting, and odynophagia. Her weight was 84.6 kg with blood pressure (BP) of 78/50. Nephrology attributed hypotension to miscalculation of dry body weight by the dialysis team. An EGD showed a 4 cm length distal esophageal ulceration. Biopsies confirmed ulcers and were otherwise negative. 

At gastroenterology office follow-up, her weight was 89.1 kg. The patient reported only occasional mild nausea and passing several medium stools daily. Gastrointestinal medications were pantoprazole, 40 mg twice daily, psyllium 18 g daily, and PEG 3350, 8.5 g every other day. Water enemas were self-administered once monthly as needed for GP symptom control.

A follow-up outpatient EGD in 2020 showed complete esophageal ulcer healing, LA grade D reflux esophagitis, and a small hiatal hernia. A tandem screening colonoscopy showed diverticulosis and small hyperplastic colon polyps.

## Discussion

In a United States (US) population-based survey of 43 million medical records from 1999 to 2014, GP prevalence was 0.16% [17]. In this study, the diagnosis of GP was considered confirmed with an impaired GE result and EGD excluding gastric outlet obstruction. Common GP symptoms may include nausea, vomiting, early satiety, upper abdominal pain, and weight loss [1-2].

Our patient ingested only one-fourth of the radioactive egg sandwich, which possibly interfered with the GE result. Additional support for the diagnosis of GP included gastric food debris noted at her initial EGD examinations and marked weight loss. While the T-½ 101 min implied mild GP, the massive weight loss (45% of body mass) and oral intake of less than 100 calories/day indicated severe disease. Also, the GE study results, at best, weakly correlate with GP symptoms [5, 9-10]. Further, with a mean 47-month follow-up and among GP patients requiring feeding jejunostomy tubes, a 32% mortality was reported by Fontana and Barnett [18]. Given the patient’s rapid clinical improvement, a follow-up GE study was considered unnecessary.

Functional dyspepsia would not account for her profound weight loss and residual gastric food debris noted at her initial EGD examinations. Failure of improvement with 10 weeks of dialysis and the 10 subsequent admissions for GP during 2014 and 2015 excluded uremic enteritis as the etiology of her illness. Normal glycated hemoglobin (HbA1C) levels ruled out poorly controlled diabetes. Laboratory and imaging results did not suggest diabetic ketoacidosis or intestinal pseudo-obstruction as causative of her illness. 

The following reports suggest that GP is part of a generalized gut dysmotility disorder [5, 7-10]. Two studies using wireless motility capsules found delayed colonic transit times in GP patients, but not in healthy diabetic subjects [7-8]. Another study using small bowel manometry found 80% of patients with suspected GP had intestinal dysmotility, but only 28% of these had an abnormal GE result [9]. In those patients with normal small bowel motility, only 6% had delayed GE; and GP symptoms severity correlated with small bowel dysmotility but not GE [9]. Also, abdominal bloating and distention are prevalent in these patients and correlate with GP illness and treatment failure [5, 10]. The study patient was not evaluated for intestinal dysmotility given her rapid improvement and as motility testing are not locally available. 

While the dietary fiber hypothesis has been largely credited to Denis Burkitt [19], his crucial observations and those by others on the association of dietary fiber with stool bulk and rapid intestinal transit have been largely forgotten [13-15]. For example, the United Kingdom (UK) Navy personnel subsisting on a typical Western fiber-depleted diet produced a mean 104 g stool daily with mean intestinal transit times of 83 hours. However, rural Ugandan villagers subsisting on their native high fiber diets had mean daily stool weights of 470 gm with mean transit times of 36 hours [13]. In this study, transit times and stool weights were not influenced by ethnicity.

As self-induced constipation can impair gastric emptying in healthy volunteers and since small stools are linked with impaired intestinal transit times, constipation could account for the above-observed findings in these patients [12]. Additionally, one study found moderate to severe constipation in most patients with symptoms of GP, and constipation severity correlated with GP symptoms [11]. 

That dietary fiber can alleviate GP illness is counterintuitive, given reports that fiber impairs GE [20-21]. However, GE studies at best weakly correlate with GP illness [5, 9-10], and these studies overlooked stool bulk and intestinal transit as independent variables that could impact study results. If increased stool bulk and/or accelerated intestinal transit does not occur when dietary fiber is introduced, then improved gastric function and GP symptoms would not be expected.

As dietary fiber has weak laxation effects, osmotic laxatives are often necessary to maintain increased stool bulk (as occurred with our patient). Moreover, as gut microflora varies widely in the general population, a broad variation in dietary fiber tolerability among patients is expected. An initial selection of the dietary fiber is often a trial-and-error process (clinical observations by co-author, JWJ). One rheological in vitro study of 10 common, commercially available soluble fiber supplements concluded that gum arabic and partially hydrolyzed guar gum (the latter was given initially to our patient) would be best tolerated by GP patients [22]. Given these concerns, an initial small dose of soluble dietary fiber was recommended and the dosage increased as tolerated. Our patient received partially hydrolyzed guar gum and psyllium, both of which are soluble fiber. Avoidance of insoluble fiber and roughage is recommended to improve GE and prevent gastric bezoars [23]. 

While dietary fiber is well-known to increase intestinal gas, those supplements altered chemically by the manufacturer (such as partially hydrolyzed guar gum and methylcellulose) are less prone to cause this issue (clinical observations by co-author, JWJ). Once excellent stool bulk is achieved, the increased intestinal gas commonly associated with other fiber supplements also tends to be less of an issue (clinical observations by co-author, JWJ). This is probably due to changes in the gut microflora and/or intestinal transit times being halved. Lactulose and sorbitol should be avoided as they can increase intestinal gas. 

Dietary fiber is defined as indigestible and non-absorbable carbohydrate polymers with at least three monomeric units [19]. In addition to its beneficial effects on stool bulking and enhanced intestinal transit, dietary fiber is a prebiotic influencing the gut microbiome with its metabolism by the intestinal flora into short-chain fatty acids.

Psyllium, 402 g/container, retails for $15. PEG 3350, 510 g retails for $13.55. The above prices were found on Amazon.com on August 4, 2021. For this patient, Konsyl® and PEG 3350 cost annually less than $1 per day. Reusable enema bags cost $20. Notably, the study patient found enemas helped prevent emergency room visits. 

There were eight fewer admissions when comparing the years 2014-2015 to 2018-2019. Using the cost analyses by Wadhwa et al. of one GP admission averaging $34,585, the estimated hospital cost savings exceeded $125,000/year when comparing the two-year time frames [24].

The multiple interventions during the April 2013 admission obscure the fundamental reason for this patient’s improvement. However, the jejunostomy tube was removed within four months of insertion and only 39% of GP patients had improvement of nausea and vomiting with jejunostomy tube insertion [18]. This patient received peritoneal dialysis for just 34 days. Notably, peritoneal dialysis only impairs GE when “full” (after dialysate is infused) but not when “empty” [25]. Thus, jejunostomy tube insertion and discontinuation of peritoneal dialysis were unlikely to be major causative factors in the patient’s marked improvement. 

The authors believe the probable cause for the case study’s marked clinical improvement is the daily dietary fiber supplementation, which was supported by osmotic laxatives and enemas on an as needed basis to achieve several medium to large stools daily. Of note, PEG 3350 dosage at the study conclusion was only 8.5 gm every other day and water enemas were once monthly as needed.

## Conclusions

This case study with severe diabetic GP had an excellent response to a low-cost treatment regime directed at increasing stool bulk, enhancing bowel motility, and mobilizing intestinal gas by using fiber supplements with as needed osmotic laxatives and water enemas. This report suggests constipation substantially contributes to GP. Prospective, controlled studies are needed.
